# Effects of Aquatic Training and Bicycling Training on Leg Function and Range of Motion in Amateur Athletes with Meniscal Allograft Transplantation during Intermediate-Stage Rehabilitation

**DOI:** 10.3390/healthcare10061090

**Published:** 2022-06-11

**Authors:** Yake Chen, Yonghwan Kim, Moonyoung Choi

**Affiliations:** 1 Department of Public Sports, Luoyang Normal University, Luoyang 471934, China; chenyake@lynu.edu.cn; 2 Department of Physical Education, Gangneung-Wonju National University, Gangneung 25457, Korea; yhkim@gwnu.ac.kr; 3 Department of Sports Science Convergence, Dongguk University, Seoul 04620, Korea

**Keywords:** aquatic training, bicycling training, meniscal allograft transplantation, rehabilitation, strength, dynamic balance, range of motion

## Abstract

Meniscal allograft transplantation (MAT) is a treatment modality for restoring knee function in patients with irreversible meniscal injury. Strengthening programs to promote functional recovery are treated with caution during the intermediate rehabilitation phase following MAT. This study analyzed the effects of aquatic training (AQT) and bicycling training (BCT) during the intermediate stage of rehabilitation in amateur athletes that underwent MAT. Participants (*n* = 60) were divided into AQT (*n* = 30) and BCT (*n* = 30) groups. Both groups performed training three times per week from 6 to 24 weeks following surgery. International Knee Documentation Committee Subjective Knee Evaluation Form (IKDC) score, knee joint range of motion (ROM), isokinetic knee strength, and Y-balance test (YBT) performance were evaluated. All measured variables for the AQT and BCT groups improved significantly after training compared with pre-training values. The IKDC score and YBT were significantly higher for AQT than for BCT. The knee flexion ROM and isokinetic muscle strength were significantly improved in the BCT group compared to those in the AQT group. The AQT group exhibited greater improvement in dynamic balance, whereas BCT provided greater improvement in isokinetic muscle strength. AQT and BCT were effective in reducing discomfort and improving knee symptoms and functions during intermediate-stage rehabilitation following MAT in amateur athletes.

## 1. Introduction

The meniscus is a fibrocartilaginous tissue that efficiently performs major knee functions such as load distribution, shock absorption, joint lubrication, and stability [1]. Damage or tears to the meniscus can cause articular cartilage degeneration and knee instability, ultimately leading to the early progression of osteoarthritis [2]. Therefore, the younger and more active the patient, the more appropriate is treatment aimed at preserving the tissue after meniscal injury [3]. Generally, meniscus repair as a surgical option to mend the damaged meniscus is preferred over meniscectomy to remove the meniscus [4]. However, suturing of the damaged meniscus is not possible in all situations. Depending on the type, extent, and location of the damage, repair of the horn is occasionally possible; in this case, meniscectomy is inevitable [5]. However, while removal of the meniscus improves pain and function in the short term, it may alter normal knee mechanics over the long term, thereby unbalancing the load on the tibiofemoral joint and increasing the risk of premature osteoarthritis [6]. Therefore, in young, active patients, meniscal allograft transplantation (MAT) may be an effective option for improving knee pain and function, improving biomechanics, and delaying osteoarthritis progression [7].

Regarding the rehabilitation protocol after MAT, previous studies have recommended performing range-of-motion (ROM) exercises as early as possible immediately after surgery [8,9]. However, considering the need for revascularization, fixation, and healing of the transplanted meniscus during the early post-surgical stage, partial weight-bearing is recommended rather than full weight-bearing for 4 to 6 weeks [10,11,12]. To minimize the posterior shear and rotational stress on the transplanted meniscus, full weight-bearing exercise is possible from 6 weeks after surgery, and it is stressed that patients should proceed slowly [8,13]. The closed kinetic chain movement that places a load on the meniscus is initially restricted; however, patients are allowed to perform light walking at 6 weeks and light running at 12 weeks after surgery [10,14].

Bicycling training (BCT) is recognized as a relatively safe load exercise during the early stages of rehabilitation for patients who underwent MAT and is commonly used to improve ROM and muscle function [14]. However, BCT does not reflect actual functional movements compared to dynamic running-based sports, and stimulation of the muscles is relatively limited [15]. Moreover, BCT is a beneficial aerobic exercise that can be performed during the early stage, but it requires more effort and time to reach the target exercise intensity because the load and fatigue are concentrated on the lower body [16]. Several previous studies have emphasized the importance of BCT in parallel with muscle strength and proprioception training for symptom improvement and functional recovery in the early rehabilitation stage following MAT [9,17].

Meanwhile, aquatic training (AQT) minimizes pain without interfering with the healing and fixation of the transplanted meniscus; AQT enables sport-specific movements, improves knee function by stimulating various muscles, and can be an effective intervention [18]. Furthermore, AQT can elicit an aerobic cardiorespiratory response similar to BCT with relatively little effort because hydrostatic pressure, the pressure exerted by water, provides load and fatigue stimuli to the entire body [19]. However, despite the positive effects of AQT, studies comparing AQT to BCT during the intermediate postoperative stage are rare.

Therefore, in this study, the subjective knee score, knee ROM, isokinetic knee strength, and dynamic balance were compared and analyzed between the AQT group conducted in water and the BCT group conducted on land during the intermediate rehabilitation stage in amateur athletes who underwent MAT. Through this study, the effectiveness of the two training methods was tested, and a better rehabilitation training method was discovered. Finally, the study was intended to contribute to the production of a safe and effective rehabilitation training program.

## 2. Materials and Methods

### 2.1. Experimental Design

This study complied with the Declaration of Helsinki and was approved by the Gangneung-Wonju National University Institutional Review Board (approval number: GWNU IRB 2021-13; approval date: 25 February 2021). Male and female amateur athletes who underwent MAT were recruited through a hospital bulletin board. Participation was voluntary, and only patients confirmed by a surgical specialist as capable of safely proceeding with the rehabilitation program were included. Recruited patients were divided into AQT and BCT groups, and the training intervention was performed. AQT or BCT was assigned through consultation and reflected the preferences of the participants. Subjective evaluations and ROM tests were performed at 6, 12, and 24 weeks after surgery. Due to safety considerations regarding the surgical site, muscle strength and dynamic balance were not tested at 6 weeks and were only measured at 12 and 24 weeks.

### 2.2. Participants

Sample sizes were calculated using G*power software (G*power 3.1, University of Düsseldorf, Düsseldorf, Germany). The main analysis method in our study was non-parametric comparison between the two groups. The setting conditions are as follows: Mann–Whitney test (two groups); effect size d = 0.5, α error probability = 0.05, and power (1-β error probability) = 0.80. The suggested sample size is 106 people.

Initially, 111 amateur athletes (88 male and 23 female, age 25–35 years) who underwent MAT were recruited. We use only male data for analysis; females are excluded. Five athletes dropped out during the study period, and the 18 women who completed training were far too few compared to men. Therefore, the female athletes were trained but were excluded from the analysis. The final analysis included 60 male athletes (AQT, *n* = 30; BCT, *n* = 30). The exclusion criteria were as follows: other lesions of the knee confirmed by radiological examination (*n* = 7), accompanying injuries such as anterior cruciate ligament rupture (*n* = 7), past knee injuries or surgery history (*n* = 6), dropouts, and patients who did not attend the final visit (*n* = 8). Athletes participated in soccer (*n* = 17), badminton (*n* = 7), tennis (*n* = 2), basketball (*n* = 6), martial arts (*n* = 5), baseball (*n* = 10), handball (*n* = 2), volleyball (*n* = 1), taekwondo (*n* = 4), wrestling (*n* = 1), judo (*n* = 2), and other sports (*n* = 3).

### 2.3. Subjective Knee Score

Knee scores related to patients’ subjective symptoms and function were measured using the International Knee Documentation Committee Subjective Knee Evaluation Form (IKDC) [20]. The IKDC consists of 18 items related to sports participation, including symptoms, functions, and activities of daily living affected by knee injury or surgery. Questions related to knee symptoms evaluated pain, stiffness, swelling, locking/catching, and giving way. Questions related to knee function and sports participation rated the level of sporting activity, ascending and descending stairs, kneeling, squatting, flexing the knee, sitting, running, jumping, starting and stopping quickly, and subjective current knee function. A score was assigned to each question according to its importance, and after summing the scores for all questions, that sum was converted according to the calculation formula to obtain a total score. The highest possible total score is 100. A total score of 100 indicates that there are no knee symptoms or functional limitations and no limitations in sports or activities of daily living. The total score conversion method for all questions is as follows:

Total score = (sum of items/maximum possible score) × 100(1)

### 2.4. Range of Motion

The ROM was measured using a manual goniometer. Each measurement was performed twice, and the average value of the measured ROM was used for analysis. The axis of joint movement was aligned with the lateral epicondyle of the femur. The stationary arm of the goniometer was aligned with the femur using the greater trochanter as a reference; the movement arm was aligned with the fibula using the lateral malleolus [21].

The knee flexion ROM was measured with the participant in the prone position. The torso was fixed, and the patient was instructed to bend the knee to the maximum, taking care not to cause any movement of the spine and pelvis. ROM recorded the endpoint as the maximum flexion angle. The knee extension ROM was measured with the participant in the supine position. A support was placed under the thigh such that the knee was fully extended without the patella touching the ground, and the foot was placed beyond the edge of the examination table. The endpoint of the ROM, at which the patient can extend the knee maximally, was recorded as the maximum extension angle.

### 2.5. Isokinetic Knee Strength

For the isokinetic knee strength test, the strengths of the extensor and flexor muscles of the knee joint were measured using an isokinetic dynamometer (Humac Norm; CSMi, Stoughton, MA, USA). The isokinetic dynamometer measures the maximum resistance of the patient through muscle contraction against a mechanically controlled constant velocity [22]. The tests were performed at angular velocities of 60°/s and 180°/s. To maintain a consistent examination posture, the participants sat in the examination chair and aligned the axis of the dynamometer with the anatomical axis of the knee and the lateral epicondyle of the femur. In addition, to minimize compensatory movements, straps were fixed around the thighs, pelvis, and torso. Measurements were performed as uniaxial contractions for the continuous extension and flexion of the knee. To help the participants understand, the test method was adequately explained, and several prior exercises were conducted. The joint ROM for flexion and extension of the knee for the examination was set from 0° to 90°, and the maximum knee extension was set to 0°. The participant was prepared while waiting for the examiner’s signal with the knee flexed at 90°.

Upon the examiner’s start signal, extension was first measured with maximum muscle contraction, and flexion was subsequently measured with maximum muscle contraction using the following protocol: The patient repeated measurements four times at an angular velocity of 60°/s and four times at an angular velocity of 180°/s. For muscle strength, peak torque (Nm) was measured. The average muscle power (W) was also measured. The absolute values of the measured muscle strength and power were divided by the patient’s body weight to obtain relative values, thereby removing differences based on body weight. Finally, to compare the muscle strength ratio of the involved and uninvolved knees between groups, the limb symmetry index (LSI) was calculated using the following formula:

LSI = (Involved limb/Uninvolved limb) × 100(2)

### 2.6. Y-Balance Test

Dynamic balance ability was measured using the Y-balance test (YBT) equipment (Y Balance Test™, Cerder Park, TX, USA) [23]. The examiner demonstrated the examination posture and sequence of movements and allowed participants adequate practice. The participants stood on one leg with the foot on the central stance plate for the examination. Then, while maintaining balance in a single-leg stance, a series of motions were performed to extend the opposite leg and push the reach indicator as far as possible with the tip of the toe in the anterior (ANT), posteromedial (PM), and posterolateral (PL) directions. Failure was considered to have occurred if the foot of the reaching limb touched the ground or when balance of the stance limb was lost. The test was conducted measuring the healthy leg first and then the operated leg. After measuring each of the three directions twice, the higher score was used in the analysis. The results were recorded in centimeters, and leg length was used to calculate the final score. Leg length was measured using a tape measure to determine the distance between the anterior superior iliac spine of the pelvis and the medial malleolus of the distal tibia. The total score for the three directions was calculated, and the LSI was compared between the groups in the same manner as muscle strength.

YBT total score = (sum of the three reach directions/3 × limb length) × 100(3)

### 2.7. Training Program

The rehabilitation program was conducted as shown in Table 1. Early-stage training was performed identically without any difference between the AQT and BCT groups. According to MAT rehabilitation guidelines, ROM and partial weight-bearing exercises were performed in the initial stage [9,24]. All participants wore a brace that kept the knee fully extended for 6 weeks after surgery, and ambulation using crutches was performed with partial weight-bearing. Passive ROM exercises to restore knee motion were started immediately after surgery. The goal was to restore the knee ROM to 0–90° for the first 2 weeks, followed by 120° for 4 weeks and 135° for 6 weeks. Immediately after surgery, isometric quadriceps contraction, straight leg raise, and active knee extension were performed to strengthen the quadriceps muscles in an open kinetic chain. In the closed kinetic chain, the participant wore a brace and performed weight shifts and calf raises with the knee fully extended. Three weeks after surgery, wall squats with a limited ROM of 60° were allowed.

Complete weight-bearing accompanied by knee flexion greater than 90° without the use of crutches and braces was allowed from 6 weeks after surgery. After 6 weeks, closed kinetic chain exercises, such as squats, lunges, and step-ups, were initiated under full weight-bearing without an orthosis, and single-leg balance was included to improve the muscular and nervous systems. At this stage, knee extension was allowed to increase the load by adding resistance, but knee flexion allowed only active motion without resistance for 12 weeks. Light running and jump-landing training were initiated 12 weeks after surgery. Light sports activities were allowed after six months, and vigorous contact sports were permitted after nine months.

#### 2.7.1. Intervention Program: Aquatic Training

The AQT program was conducted 3 times per week by applying the continuous water aerobic routine (CWAR) described in the study by Kruel et al. [25]. The CWAR comprised eight routines, in which four water aerobic exercises (stationary running, cross-country skiing, jumping jacks, and frontal kicks) were each repeated twice. Each routine was performed continuously for 4 min without an interval. Therefore, the total training time was 32 min. All lower body movements were performed simultaneously with bilateral arm push–pulls for whole-body exercise. The training intensity of the AQT program was controlled using Borg’s rating of perceived exertion (RPE) scale and an electronic heart-rate-monitoring device (Polar H10, Polar Electro, Bethpage, NY, USA). Verbal scales were used to express the effort level on a scale of 13 to 15 [26].

The examiner trained participants on the standard guidelines of the RPE scale to aid participants in verbally expressing their perceived level of effort as accurately as possible. In addition, adequate practice was performed before the actual training to familiarize participants with the feelings corresponding to minimum and maximum effort. Participants performed the routines as directed by the instructor at intensity levels corresponding to ‘somewhat hard, 13’ to ‘hard, 15’. The suggested heart rate exercise intensity was 60–75% of the maximum heart rate.

#### 2.7.2. Intervention Program: Bicycling Training

The BCT program was conducted 3 times per week, similar to the AQT, following the training intensity and duration of the continuous bicycling program proposed in a previous study [27]. A stationary friction-loaded cycle ergometer (Monark Model 864, Monark Crescent AB, Varberg, Sweden) was used for training. The saddle height of the stationary bicycle ergometer was individually adjusted based on the participant’s body structure such that one leg was extended to a maximum of ~ 25° when the participant was sitting on the saddle [28] The BCT group performed continuous cycling for 32 min at an intensity of 60–75% of maximum heart rate while trying to maintain a pedaling speed of 60 RPM. To control the exercise intensity of the participants during training, the heart rate change was monitored in real time using an electronic heart-rate-monitoring device (Polar H10, Polar Electro, Bethpage, NY, USA), as was done in the AQT group.

### 2.8. Statistical Analysis

Data analysis was performed using SPSS Statistics (version 25.0; IBM Corp., Armonk, NY, USA). The normality test of the main variables was performed using Kolmogorov–Smirnov and Shapiro–Wilk tests. Since the variables did not exhibit a normal distribution, we performed a non-parametric analysis. Continuous variables are expressed as means and standard deviations, and non-continuous variables are expressed as numbers and percentages of patients. The Kruskal–Wallis test and the post hoc Bonferroni test were used for within-group tests of IKDC and ROM, which were tested three times. The Wilcoxon signed-rank test was used for comparison pre- and post-training within groups of twice-tested strength and YBT. Additionally, the Mann–Whitney U test was performed for between-group comparisons. The significance level was set at *p* < 0.05.

## 3. Results

### 3.1. General Characteristics of Participants

The participants were classified according to the intervention group, and their general characteristics are summarized in Table 2. When the AQT and BCT groups were compared, there were no statistically significant differences between the groups regarding age, height, weight, body mass index, injury site, or dominant side.

### 3.2. Subjective Knee Score

Table 3 shows the differences in IKDC scores analyzed by group and measurement week to evaluate the subjective knee score after MAT. Both the AQT and BCT groups exhibited significantly improved IKDC scores over time following surgery (*p* < 0.001 and *p* < 0.001, respectively). There was no significant difference at 6 and 24 weeks in the comparison between groups for each week, but at 12 weeks, the AQT group achieved significantly higher IKDC scores than the BCT group (*p* = 0.033).

### 3.3. Knee Range of Motion

Table 4 shows the changes according to group and weeks post-surgery of knee ROM after MAT. Both the AQT and BCT groups showed significant improvement in flexion (*p* = 0.010 and *p* = 0.009, respectively) and extension ROM over time following surgery (*p* = 0.012 and *p* = 0.005, respectively). In the between-group comparison for each week, there was no significant difference in flexion ROM at 6 and 24 weeks, but at 12 weeks, the BCT group showed significantly greater ROM than the AQT group (*p* = 0.015). There was no significant between-group difference regarding extension at any number of weeks.

### 3.4. Isokinetic Knee Strength

Table 5 shows isokinetic knee strength according to group and weeks after MAT. At an angular velocity of 60°/s, muscle strength improved at 24 weeks compared to measured values at 12 weeks in both the AQT and BCT groups. Similarly, at an angular velocity of 180°/s, extension and flexion of the knee in the AQT and BCT groups improved at 24 weeks compared to the 12-week values. The BCT group exhibited significantly higher LSI than the AQT group in both extension and flexion strengths at week 12 at an angular velocity of 60°/s. However, at an angular velocity of 180°/s, there was no significant difference in LSI between the groups in either extension or flexion.

### 3.5. Y-Balance Test

Table 6 shows the changes in YBT after MAT. In the involved knee of the AQT and BCT groups, the YBT direction and total score were significantly improved at 24 weeks compared to the corresponding values at 12 weeks. In the intergroup comparison of LSI, the AQT group had significantly higher scores than the BCT group at 12 weeks in all directions, as well as higher total scores. However, there were no significant differences between the groups at 24 weeks.

## 4. Discussion

AQT and BCT are partially weight-bearing and non-weight-bearing, respectively, thereby reducing the weight on the knee. AQT is often used in rehabilitation because of the hydrodynamic properties of water, including buoyancy and water pressure [29]. In addition, the stationary bicycling ergometer is unlikely to pose a risk to the recovering tissue, as it biomechanically provides controlled movement during flexion and extension [9]. In this study, a modified weight-bearing environment was provided, and the effects were compared during intermediate-stage rehabilitation of patients who had undergone MAT.

The subjective knee score evaluated by the IKDC improved significantly after training in both the AQT and BCT groups. The AQT group exhibited significantly greater improvement than the BCT group at week 12, which translates to relatively rapid recovery in the AQT group. In a similar study, the effect of early improvement was analyzed using minimal clinically important differences (MICDs). In the study by Liu et al. [30], the MICD of the IKDC score that can be applied to evaluate the outcome of patients who received MAT was proposed as 9.9. Compared to 6 weeks after surgery, at 12 weeks, the IKCD score of the AQT group increased by 16.9 points to achieve a significant MICD, whereas the BCT group showed an increase of 9.4, which was slightly insufficient for achieving an MICD.

The greater improvement exhibited by the AQT group at 12 weeks means that knee symptoms such as pain, stiffness, and swelling were significantly ameliorated by AQT compared to the results obtained by BCT. Water provides resistance such as turbulence and hydrostatic pressure while simultaneously reducing weight-bearing due to buoyancy, which may have had a combined effect [29]. In a study investigating the characteristics of water, AQT was reported to reduce pain and swelling, and promote recovery from fatigue by increasing blood circulation [31]. Therefore, the physical properties of AQT may have aided recovery after surgery, leading to physiological changes and subjective improvement of the knee condition.

Patients who have undergone MAT should be careful not to generate posterior shear and rotational forces for stable fixation and healing of implants during the early stage of rehabilitation. Therefore, the range of knee flexion is limited along with weight-bearing, and absolute immobilization is performed for at least three weeks [14]. After absolute immobilization, it is important to restore the ROM [32]. In a non-weight-bearing state, passive ROM has limitations in promoting active muscle contraction and mechanoreceptor activation; therefore, it is difficult to restore the functional movement patterns required in sports [33].

The results of this study showed that both the AQT and BCT groups displayed significantly improved knee flexion and extension ROM at 12 and 24 weeks, and the BCT group showed significantly greater knee flexion ROM than the AQT group at 12 weeks. This could be because the AQT program comprises various movements of the extremities, whereas the BCT program entails continuous pedaling involving repeated knee flexion and extension [34]. Training performed in the AQT environment is known to provide an advantage in ROM recovery, as there is less stress on the joints, owing to buoyancy resulting from the hydrodynamic properties of the aquatic medium [19].

Recovery of weakened knee muscle strength after MAT is an important factor in determining the success of postoperative outcomes and returning to sports [9]. In a study examining the long-term outcomes of patients who received MAT, decreased levels of function and activity were associated with decreased maximal quadriceps muscle strength [35]. In this study, the defect rate was evaluated using the LSI value, which is a reference value used to assess the involved side compared to the uninvolved side. In this study, both the AQT and BCT groups showed significantly improved muscle strength and achieved more than 90% on the LSI at 24 weeks. These results suggest that the inclusion of AQT and BCT in the rehabilitation process is an effective intervention for improving muscle function in patients with MAT. However, in the weekly comparison, the BCT group showed a significantly higher LSI than the AQT group at 12 weeks at an angular velocity of 60°/s. These results suggest that the intervention effect was faster for BCT than that for AQT because BCT was concentrated on the lower extremities.

The meniscus generates sensorimotor information necessary to control the stabilizing muscles surrounding the knee, as well as mechanical stability in the knee joint [36]. Free nerve endings (nociceptors) and three types of mechanoreceptors (Ruffini endings, Pacinian corpuscles, and Golgi tendon organs) exist in the anterior and posterior horns of the meniscus and the peripheral two-thirds of the body and are responsible for proprioception in the knee joint [37]. Proprioceptors transmit signals to the central nervous system (CNS) resulting from physical stimuli, such as tension or compression forces applied to the knee joint, thereby helping to regulate reflex responses and muscle coordination related to postural stability [38]. Thijs et al. [37] observed that patients with meniscal removal had significant deficits in proprioception of the knee joint and reported that MAT may contribute to restoring the reactivity of damaged knee proprioceptors. However, restoration of proprioceptive function alone cannot achieve complete improvement in postural stability, which can be further facilitated by training interventions performed during the rehabilitation stage [37]. In this study, the effects of AQT and BCT on the improvement of postural stability after MAT were evaluated using the YBT. The YBT is currently the most commonly used measurement tool for assessing the dynamic balance of the lower extremities [23]. Dynamic balance refers to the ability to maintain postural stability while moving the body or changing the position of a limb and is an important component of most daily life and sports activities [39]. AQT and BCT significantly improved the YTB results in this study. At 12 weeks, the AQT group showed significantly higher dynamic balance ability than the BCT group. Training in an AQT environment may induce instability that alters information in the somatosensory system under the influence of water turbulence, an intrinsic property of the environment [29]. In this study, unlike the BCT group, the AQT group was continuously affected by aquatic perturbation caused by water turbulence throughout the training session. This perturbation is believed to provide an additional balance stimulus for participants, such as further activation of the neuromuscular muscles of the ankle and knee joints, to restore balance. As a result, it is believed that the dynamic characteristics of AQT may have improved results earlier compared to the BCT group.

Based on the results of this study, realistic suggestions can be made for athletes who underwent MAT. If an underwater facility is available, we recommend a combination of AQT and BCT after MAT. In addition, if BCT is mainly performed without AQT, more careful observation is required to ensure that the recovery of dynamic balance is not delayed.

Based on the results of this study, it is recommended to use both AQT and BCT for athletes who have undergone MAT, but because this study was conducted without a control group, more research is needed to provide scientific evidence. This study had additional limitations. A control group was not established due to ethical considerations. Muscle strength, balance, and subjective satisfaction are possible parts of recovery over time after MAT. However, limiting rehabilitation training for research purposes to people visiting rehabilitation centers can be an ethical issue. In addition, assignment of AQT and BCT was not random because the training method chosen was based on the preference of the athlete. In particular, the AQT reflected individual preferences because it required the use of swimsuits. This study was conducted at a single rehabilitation center, there were relatively few participants, and the participants specialized in various sports; therefore, the influence of variable characteristics cannot be excluded. Analyzing only male data is one of the major limitations. In the future, it will be necessary to conduct a study by recruiting more female participants who underwent MAT. Although YBT was used for dynamic balance in this study, experiments with neuromuscular control through one-leg stabilometric measurement should be performed in future studies.

## 5. Conclusions

AQT and BCT after MAT improved subjective knee score, knee joint range of motion, isokinetic knee strength, and YBT at 24 weeks compared to pre-training values. Interim measurements performed 12 weeks after the intervention revealed that subjective knee score and YBT were higher in the AQT group, and flexion ROM and isokinetic knee strength were higher in the BCT group. Therefore, AQT and BCT with reduced weight-bearing could be effective training interventions to overcome challenges and improve symptoms and functions during the intermediate rehabilitation stage of MAT.

## Figures and Tables

**Table 1:** Rehabilitation protocol.

	1–2 Weeks	3–4 Weeks	5–6 Weeks	7–8 Weeks	9–12 Weeks	3–6 Months
Brace	O	O	O			
Crutch	O	O	O			
Weight-bearing						
1/4 of body weight	O					
2/4 of body weight		O				
3/4 of body weight			O			
Full				O	O	O
Range of motion						
0–90°	O					
0–120°		O				
0–135°			O			
Stretching						
Hamstring, Quadriceps, GCM, ITB	O	O	O	O	O	O
Strengthening						
Quadriceps sets, Straight leg raise	O	O	O			
Active knee extension	O	O	O			
Active knee flexion				O	O	
Heel raises	O	O	O			
Wall squat			O			
Squat, Lunge, Step-ups				O	O	O
Leg press machine				O	O	O
Leg extension machine				O	O	O
Leg curl machine						O
Proprioception training						
Weight shift	O					
Tandem stance		O				
Single leg balance			O			
Single leg balance with leg swing				O	O	O
Single leg squat				O	O	O
Running, Jump-landing						O

GCM, gastrocnemius; ITB, iliotibial band.

**Table 2:** General characteristics of participants.

Variables	AQT (*n* = 30)	BCT (*n* = 30)	*t* or χ^2^	*p*-Value
Age, years	28.7 ± 3.8	29.1 ± 4.0	−1.391	0.412
Height, cm	173.8 ± 2.9	174.1 ± 3.7	0.279	0.774
Weight, kg	68.7 ± 4.6	69.3 ± 5.8	0.573	0.615
BMI, kg/m^2^	22.7 ± 1.5	22.9 ± 1.7	0.384	0.631
Involved side, *n* (%)				
Right	17 (56.7%)	16 (53.3%)	0.384	0.551
Left	13 (43.3%)	14 (46.7%)		
Dominant side, *n* (%)				
Right	25 (83.3%)	23 (76.7%)	0.207	0.415
Left	5 (16.7%)	7 (23.3%)		
Involved meniscus site, *n* (%)				
Medial	9 (30.0%)	11 (36.7%)	0.211	0.258
Lateral	21 (70.0%)	19 (63.3%)		

AQT, aquatic training; BCT, bicycling training; BMI, body mass index.

**Table 3:** Subjective knee score according to group and weeks.

Variables	Weeks	AQT (*n* = 30)	BCT (*n* = 30)	*t*	*E.S*	*p*-Value
IKDC score	6	65.5 ± 15.1	63.4 ± 14.4	2.631	0.142	0.512
	12	82.4 ± 17.9 ^a^	72.8 ± 15.7 ^a^	1.346	0.570	0.033
	24	93.1 ± 11.3 ^b,c^	95.4 ± 13.2 ^b,c^	−0.831	0.187	0.794
	*p*	<0.001 *	<0.001 *			

* *p* < 0.05; IKDC, International Knee Documentation Committee; ^a^: 6 weeks vs. 12 weeks; ^b^: 12 weeks vs. 24 weeks; ^c^: 6 weeks vs. 24 weeks.

**Table 4:** Knee range of motion according to group and weeks.

Variables	Weeks	AQT (*n* = 30)	BCT (*n* = 30)	*t*	*E.S*	*p*-Value
Flexion (degree)	6	115.2 ± 8.1	113.3 ± 7.6	−3.379	0.241	0.651
	12	120.6 ± 5.4 ^a^	132.0 ± 6.5 ^a^	−1.080	1.907	0.015
	24	135.0 ± 3.9 ^c^	134.9 ± 3.5 ^c^	0.191	0.026	0.485
	* *p*	0.010	0.009			
Extension (degree)	6	10.4 ± 3.5	9.3 ± 3.1	−0.824	0.332	0.684
	12	2.1 ± 0.9 ^a^	1.1 ± 0.9 ^a^	−986	1.111	0.123
	24	−1.6 ± 0.7 ^c^	−2.0 ± 0.8 ^c^	−4.541	0.532	0.115
	* *p*	0.012	0.005			

* *p* < 0.05; AQT, aquatic training; BCT, bicycling training; E.S, effect size; ^a^: 6 weeks vs. 12 weeks; ^c^: 6 weeks vs. 24 weeks.

**Table 5:** Isokinetic knee strength according to group and weeks.

Variables	Weeks	AQT (*n* = 30)		LSI (%)	BCT (*n* = 30)		LSI (%)	Inter–Group LSI *p*-Value
		Uninvolved	Involved		Uninvolved	Involved		
60°/s extension	12	243.3 ± 45.7	105.3 ± 45.3	43.2 *	239.1 ± 38.0	127.1 ± 45.6	52.3 *	0.003
	24	258.4 ± 56.5	237.2 ± 49.2	91.9	260.3 ± 48.1	245.3 ± 51.9	94.2	0.350
	*p*	0.025	<0.001		0.021	<0.001		
60°/s flexion	12	136.2 ± 21.9	100.1 ± 24.7	73.5 *	130.0 ± 28.3	112.4 ± 30.8	86.2 *	0.005
	24	149.0 ± 22.1	136.3 ± 24.0	91.3	151.9 ± 20.8	140.4 ± 25.1	92.7	0.102
	*p*	0.014	<0.001		0.017	<0.001		
180°/s extension	12	145.3 ± 31.0	102.7 ± 39.7	70.3 *	142.6 ± 32.4	109.0 ± 35.1	76.8 *	0.213
	24	150.4 ± 29.7	142.4 ± 31.6	94.7	154.7 ± 30.6	145.9 ± 34.0	94.2	0.109
	*p*	0.210	<0.001		0.330	<0.001		
180°/s flexion	12	97.9 ± 19.9	90.4 ± 15.3	92.8	100.6 ± 11.4	98.5 ± 15.7	98.0	0.067
	24	110.1 ± 12.1	103.0 ± 10.8	93.6	115.8 ± 12.5	108.4 ± 16.4	93.9	0.153
	*P*	0.140	<0.001		0.417	<0.001		

* *p* < 0.05; AQT, aquatic training; BCT, bicycling training; LSI, limb symmetry index.

**Table 6:** Y-balance test according to group and weeks.

Variables	Weeks	AQT (*n* = 30)		LSI (%)	BCT (*n* = 30)		LSI (%)	Inter-Group LSI *p*-Value
		Uninvolved	Involved		Uninvolved	Involved		
ANT	12	62.8 ± 9.8	48.3 ± 12.3	77.4 *	61.5 ± 9.9	38.4 ± 11.0	62.3 *	0.007
	24	65.5 ± 9.3	60.5 ± 13.4	92.3	63.4 ± 11.3	59.7 ± 12.9	93.7	0.651
	*p*	0.121	<0.001		0.231	<0.001		
PM	12	80.3 ± 12.5	60.4 ± 16.3	75.0 *	74.3 ± 13.8	49.3 ± 13.6	66.2 *	0.003
	24	81.6 ± 11.8	79.6 ± 15.3	97.5	80.3 ± 12.3	72.0 ± 14.1	90.0	0.591
	*p*	0.110	<0.001		0.210	<0.001		
PL	12	78.9 ± 13.8	63.6 ± 11.4	80.8 *	75.1 ± 12.9	51.3 ± 12.9	68.0 *	0.002
	24	82.3 ± 12.4	78.6 ± 14.3	95.1	81.4 ± 13.0	76.1 ± 13.4	93.8	0.794
	*p*	0.257	<0.001		0.098	<0.001		
Total	12	86.5 ± 11.2	67.6 ± 12.1	77.7 *	82.3 ± 11.6	54.0 ± 12.3	65.7 *	0.005
	24	89.9 ± 10.3	85.1 ± 11.6	95.2	87.9 ± 11.9	81.7 ± 13.1	92.7	0.510
	*p*	0.150	<0.001		0.254	<0.001		

* *p* < 0.05, AQT: aquatic training, BCT: bicycling training, LSI: limb symmetry index, ANT: anterior, PM: posteromedial, PL: posterolateral.

## Data Availability

The data are not publicly available due to privacy or ethical reasons.
